# Awareness of Diabetic Retinopathy Among Diabetic Patients in King Khalid Eye Specialist Hospital, Riyadh, Saudi Arabia

**DOI:** 10.7759/cureus.30458

**Published:** 2022-10-19

**Authors:** Abdullah H Alhamoud, Mohammed Bajahzer, Mohammed Alshahrani, Mohammed Alghamdi, Saud Alaklabi, Hassan Aldhibi

**Affiliations:** 1 General Pediatrics, King Fahad Central Hospital, Jazan, SAU; 2 Clinical Nutrition, Applied Medical Sciences, Jazan University, Saudi Arabia, Jazan, SAU; 3 Laboratory Medicine, King Khaled Eye Specialist Hospital, Riyadh, SAU; 4 Laboratory Medicine, Medical City King Saud University, Riyadh, SAU; 5 Pediatric Medicine, King Saud Medical City, Riyadh, SAU; 6 Ophthalmology, King Khalid University Hospital, Riyadh, SAU

**Keywords:** optometrist checkup, saudi arabia, riyadh, king khalid eye specialist hospital, awareness, diabetic retinopathy, diabetic mellitus

## Abstract

Introduction: Diabetic retinopathy (DR) is a leading cause of blindness. Over a third of the population with diabetes (DM) develop DR. Therefore, we aimed to investigate the awareness level of DR in the subjects with DM attending clinics at King Khaled Eye Specialist Hospital (KKESH) in Riyadh, Saudi Arabia.

Methods: A cross-sectional study was carried out in KKESH. Responses from 360 subjects (men and women) with DM were collected using a validated questionnaire after obtaining informed consent. The questionnaire was constructed to assess the level of knowledge about DR, its screening, prevention, and treatment. The questionnaire responses were utilized thereafter to assess the association between the level of awareness of DR and the characteristics of the subjects. Pearson's chi-squared test was computed to exhibit the association and a p-value of <0.05 was considered significant.

Results: Almost all the subjects answered six or more questions, thereby considered aware of DR. Only 3.6% of the subjects knew how often their eyes should be screened for DR. Only 3.2% of the subjects with DM type 1 (DM1) knew when their eyes should be examined after being diagnosed with DM. Men exhibited a significantly higher (p= 0.04) level of awareness compared to women. Comparing the other characteristics of the subjects exhibited no significant differences. In conclusion, although the studied population exhibited a good level of awareness about DR, they would benefit from further education on DR, especially on how regularly their vision is recommended to be examined to prevent DR and its consequences.

## Introduction

Diabetes mellitus (DM) is a chronic, endocrine, metabolic disease characterized by an impaired capacity to utilize plasma glucose, secondary to the lack of insulin secretion, or provoked insulin balance. The prevalence of DM is increasing all over the globe [1]. Considering such projections, DM would persist in being a major public health burden [2]. The ophthalmological complications in subjects with DM are a common cause leading to blindness globally [3]. Diabetic retinopathy (DR) is a serious complication of DM, which damages the capillaries and nerves of the eyesight due to uncontrolled blood glucose. The risk of developing DR increases over time as it occurs in approximately 77% of diabetic cases after 10 years of DM onset [4] and reaches 100% after 30 years [5]. This is relevant to the approximately 24% prevalence among the Saudi population ranging between 20 and 79 years of age in 2013 [6]. In fact, studies from different regional hospitals in Saudi Arabia reported a comparable prevalence of DR in their assessed populations; 36.4% in the southern region of Saudi Arabia [7], 36.1% in Taif [8], and 34.5% in the Al-Madinah region [9]. The risk to develop complications related to DM is four-fold higher in subjects with DM who lack the awareness of such anomalies [10]. Thus, educating such subjects properly about DM and its possible complications would raise their awareness to adopt preventive measures, report complications earlier, and at a public scale would limit the prevalence of DM complications, such as DR [11]. The prevalence of DM and DR are consistently growing in Saudi Arabia, thereby their health and economic burden on the care system and population would be greater. Thus, we aimed to assess the level of awareness of DR and its related risk factors among the subjects with DM attending King Khalid Eye Specialist Hospital (KKESH) clinics in Riyadh, Saudi Arabia.

## Materials and methods

A cross-sectional study was designed and conducted in Riyadh, at KKESH, between January 1 and July 31, 2020. The study targeted subjects with DM attending KKESH. Subject data were obtained through the hospital medical registry and eligible individuals were contacted for recruitment. A total of 1550 eligible individuals were contacted, among which 360 were willing to participate and were included in the study. The included 360 subjects were 239 male and 121 female, aged between 24 and 85 years. Responses about socioeconomic data, age, gender, type of DM, duration of DM, type of medication, and marital status were obtained using an adapted version of the questionnaire that was used in a previously published study [12]. The questionnaire comprised 10 questions: three about DR knowledge, five about DR screening, and two about DR prevention and treatment (Table 1). Subjects who correctly answered six or more questions were considered aware of DR.

**Table 1:** Awareness Questionnaire of Diabetic Retinopathy DM: diabetes mellitus; DR: diabetic retinopathy

Question	Answer		
Do you think there is a relationship between retinopathy and DM?	Yes		No
Do you think diabetes mellitus may lead to blindness?	Yes		No
Have your eyes been checked by a doctor last year?	Yes		No
Do you think good control of diabetes might prevent DR?	Yes		No
Can a diabetic patient have eye problems at the same time as a diabetes diagnosis? DMI	Yes		No
Can a diabetic patient have eye problems at the same time as a diabetes diagnosis? DMII	Yes		No
How frequently should a person with diabetes undergo an eye checkup?	Only when vision is affected	Yearly or every two years	Every six months
When you are diagnosed with diabetes for the first time, you must screen your eye	Only if there are eye symptoms	Five years after diabetes diagnosis	At the time of diabetes diagnosis
Do you think retinopathy is a treatable condition?	Yes		No
Do you think seeing an optometrist (regular eyeglass store) is enough for people with diabetes?	Yes		No
No need for the regular screen for DR if both eyes are good?	Yes		No

Statistical analysis

All the obtained data were managed and analyzed using IBM SPSS Statistics for Windows, Version 22.0 (Released 2013; IBM Corp., Armonk, New York, United States). The general characteristics and questionnaire responses were computed using frequencies. Pearson's chi-squared test was utilized to assess the association between the subjects’ subgroups and their questionnaire responses. The level of significance was considered at a p-value of <0.05.

Ethical consideration 

This study was reviewed and got approval from the institutional review board in KKESH, RP 2012-P (reference number is RD/26001/IRB/0152-20). The consent details and forms were explained to all participants for approval before directing them to the questionnaire. The questionnaire was answered by all the participants anonymously throughout the study period to maintain the confidentiality of the participant's data.

## Results

Responses from 360 subjects were utilized for the analysis in the current study. The general characteristics of the subjects are shown in Table 2. Most of the subjects were males (66.4%), 40-65 years old (69.4%), married (81.7%), diagnosed with DM2 (91.4%), with a history of DM that exceeds 10 years (89.2%), and using insulin treatment for DM (64.7%) (Table 2).

**Table 2:** General Characteristics of Participants DM: diabetes mellitus

Characteristics			
Gender			
Male	Female		
239 (66.4%)	121 (33.6%)		
Age			
<40 years	40-65 years	>65 years	
27 (7.5%)	250 (69.4%)	83 (23.1%)	
Marital Status			
Married	Single	Other	
294 (81.7%)	15 (4.2%)	51 (14.2%)	
Diabetes Type			
DM1	DM2		
31 (8.6%)	329 (91.4%)		
History with diabetes			
<5 years	5-10 years	>10 years	
10 (2.8%)	29 (8.1%)	321 (89.2%)	
Medication			
Insulin	Tablet	Insulin + tablet	NA
233 (64.7%)	59 (16.4%)	66 (18.3%)	2 (0.6%)

Correctly answering at least six of the questionnaire questions was considered the threshold of awareness in the current study. Only five subjects (1%) answered less than six questions correctly compared to 355 subjects (99%) who satisfied the awareness criteria. More than 90% of the subjects believed that there is a relationship between DM and retinopathy, DM may lead to blindness, and that good control of DM may prevent DR (Table 3).

**Table 3:** Awareness Questionnaire of Diabetic Retinopathy with the Percentage of Correct Answers DM: diabetes mellitus; DR: diabetes retinopathy

Questions		Correct answer	%
Q1	Do you think there is a relationship between retinopathy and DM?	Yes	98.3
Q2	Do you think diabetes mellitus may lead to blindness?	Yes	91.4
Q3	Have your eyes been checked by a doctor last year?	Yes	86.1
Q4	Do you think good control of diabetes might prevent DR?	Yes	96.7
Q5	Can a diabetic patient have eye problems at the same time as a diabetes diagnosis? DMI	No	51.6
	Can a diabetic patient have eye problems at the same time as a diabetes diagnosis? DMII	Yes	79.3
Q6	How frequently should a person with diabetes undergo an eye checkup?	Yearly or every two years	3.6
Q7	When you have diabetes for the first time, when must you screen your eye? DMI	Five years after the diabetes diagnosis	3.2
	When you have diabetes for the first time, when must you screen your eye? DMII	At the time of diabetes diagnosis	88.8
Q8	Do you think retinopathy is a treatable condition?	Yes	92.8
Q9	Do you think seeing an optometrist (regular eyeglass store) is enough for people with diabetes?	No	97.2
Q10	No need for the regular screen for DR if both eyes are good?	No	93.9

Similarly, more than 90% of the subjects believed that DR is treatable, optometrist checkup is not enough for people with DM, and regular screening for DR is necessary even if both eyes are good (Table 3). Only 3.6% of the subjects thought that a patient with DM should undergo an eye checkup annually or every two years. Questions 5 and 7 of the questionnaire varied in their level of correct responses as they were specific to the types of DM; 79.3% of the subjects with DM2 correctly answered question 5 compared to 51.6% of the subjects with DM1. Only 3.2% of the subjects with DM1 answered question 7 correctly compared to 88.8% of the subjects with DM2. The level of awareness was significantly higher (p = 0.04) in males compared the females (Table 4).

**Table 4:** Awareness Analysis DM: diabetes mellitus

	Aware	Unaware	p Value
Gender			
Male	99.6%	0.4%	0.045
Female	96.7%	3.3%	
Diabetes Type			
DM1	93.5%	6.5%	0.06
DM2	99.1%	0.9%	
Age			
<40	96.3%	3.7%	0.48
40-65	98.8%	1.2%	
>65	98.8%	1.2%	
History with diabetes			
<5	100%	0.0%	0.44
5-10	96.6%	3.4%	
>10	98.8%	1.2%	
Medication			
Insulin	99.1%	0.9%	0.16
Tablets	96.6%	3.4%	
Both	98.5%	1.5%	
Marital status			
Single	93.3%	6.7%	0.21
Married	98.6%	1.4%	
Other	100%	0.0%	

**Figure 1:**
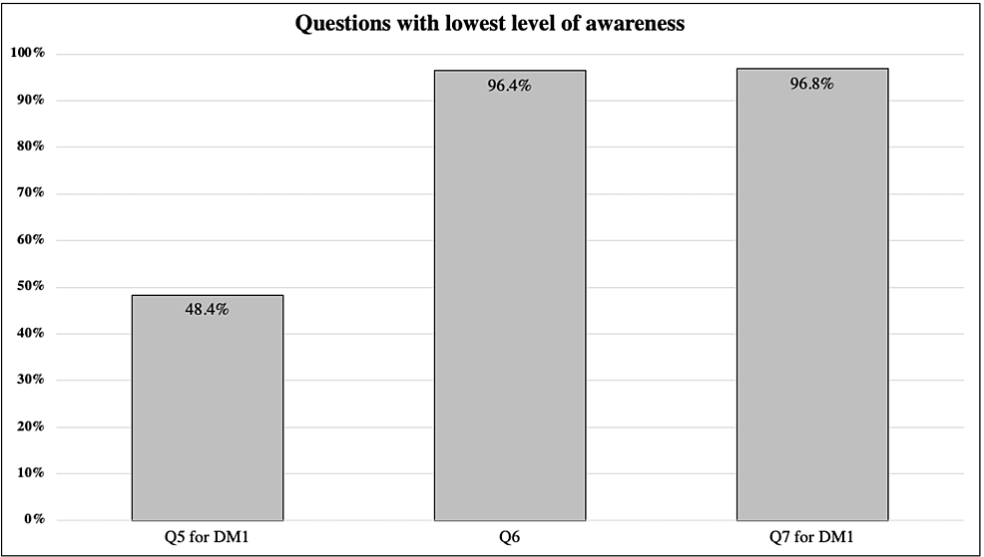
Questions With the Lowest Level of Awareness Q: question; DM: diabetes mellitus

There were no significant differences in the awareness level when the DM types, age groups, history with DM, medications, and marital status were compared (Table 4). In general, a higher percentage of subjects with DM1 incorrectly answered the questions with the lowest level of awareness (Figure 1).

## Discussion

In the current study, most of the subjects with DM2 were aware of their eye's annual examination at the time of diagnosis. On the contrary, most subjects with DM1 were not aware of the eye's annual examination, which should be done five years after being diagnosed. This would be difficult to compare with other published studies, given the different assessments of DR knowledge utilized in the studied populations. However, in Saudi Arabia and other middle eastern populations, several studies have estimated the prevalence of DR as 30-40%. Furthermore, a steady increase in the number of DR cases reported highlighted the importance of promoting awareness about DM, DR, and their complications [8,13,14]. Studies reported on DM2 in India showed a poor control of DM has a higher risk for DM complications [1,15]. 

Comparing the outcomes of our current study with a recent study published in Riyadh, the current study showed a higher level of awareness among our population compared to the population of the previous study; 98.3% in the current study versus 88% in the previous study were aware of the link between DM and DR, 96.7% versus 76% were aware of the preventive role of controlling DM to reduce the risk for DR, and 91.4% versus 66% were aware of the causal link between DR and blindness [16]. 

The awareness level of the population in the current study (93.5% in DM1 and 99.1% in DM2) is in fact higher compared to the awareness of populations from studies in other regions in Saudi Arabia and some neighboring countries. Locally, for instance, the reported level of awareness in Hail and Al Jouf together and in Jeddah alone was 76% and 83%, respectively [7,12]. In neighboring countries, the level of awareness was fairly high: 93% in Oman [17] while an awareness level of 88% was reported in both Jordan [18] and Turkey [19]. The level of awareness of the consequences of DM on vision and eyes varies worldwide. In Switzerland, for instance, the level of awareness is high (96%) [20] and almost comparable to the level of awareness we observed in the current study. However, the awareness level in Malaysia is somewhat average (86%) [21]. Furthermore, a study in the rural Tamil Nadu area of India reported a low level of awareness of (74%) of DM and the consequent risk for DR [22]. Nonetheless, it is important to note that we evaluated the awareness of DR in subjects with DM1 and DM2, while most of the other mentioned studies evaluated the awareness in DM2 only, which in part could explain the heterogeneity of results.

Given that the development of DR follows a silent course of changes before manifestation into a disease, the routine vision and eye examination serves the purpose of diagnosing DR early or preventing its onset and complications in the population with DM. This particular recommendation should be emphasized in the population of the current study. Although 86.1% of the population underwent an ophthalmologist checkup during the previous year, only 3.6% of these knew how regularly such checkups should be carried out. This proportion is quite worrisome, especially when it is compared to Switzerland's population among which 97.5% knew how regularly they should get their vision checked [20]. 

One strength of the current study is the relatively large population compared to previous studies. An additional strength is the fact that we explored both awareness and practices regarding DR in a population-based study including subjects with DM1 and DM2, whereas previously such assessments had been carried out mostly in populations with DM2.

However, some limitations in the current study should be considered. We obtained and analyzed data using a self-reported method that is prone to recall bias. Another potential limitation is, selecting a population at the ophthalmologic center, which could exaggerate the level of awareness measured and thereby undermine the level of awareness in the general population with DM. Still, the current population shares similar characteristics with the populations in other local studies [7,12]. Although the generalizability of the current results is limited, evaluating data of facilities specialized in certain areas of medical care is important to evaluate patients’ care and would provide a practical example for other care centers, e.g., primary care facilities, to improve their DM/DR care, education, and awareness services. 

## Conclusions

In conclusion, the study showed a high level of awareness of DR and its impact on vision, and its link to uncontrolled DM among subjects with DM1 and DM2. However, a lack of knowledge among both types of DM (only a few knew how regularly they should get their vision checked) and in subjects with DM1 (only a few knew when they should get screened for eye complications after being diagnosed with DM) should be highlighted and DM/DR care, education, and awareness services would benefit from the emphasis on these recommendations. Limited data available on the level of awareness of DR in subjects with DM1 merits further study among such populations.
